# Amputation stump perfusion is predictive of post-operative necrotic eschar formation

**DOI:** 10.1016/j.amjsurg.2018.05.007

**Published:** 2018-05-19

**Authors:** Gayan S. De Silva, Khalid Saffaf, Luis A. Sanchez, Mohamed A. Zayed

**Affiliations:** a Washington University School of Medicine, Department of Surgery, Section of Vascular Surgery, St. Louis, MO, USA; b Veterans Affairs St. Louis Health Care System, St. Louis, MO, USA

**Keywords:** Amputation, Stump perfusion, Eschar, Fluorescence angiography

## Abstract

**Background:**

A large proportion of patients develop poor amputation stump healing. We hypothesize that Laser-Assisted Fluorescent Angiography (LAFA) can predict inadequate tissue perfusion and healing.

**Methods:**

Over an 8-month period we reviewed all patients who underwent lower extremity amputation and LAFA. We evaluated intra-operative LAFA global and segmental stump perfusion, and post-operative modified Bates-Jensen (mBJS) wound healing scores.

**Results:**

In 15 patients, amputation stumps with lower global perfusion demonstrated higher mBJS (*P* = 0.01). Lower suture-line perfusion also correlated with more eschar formation (*P* < 0.001). Diabetic patients had higher mBJS (*P* = 0.009), lower stump perfusion (*P* = 0.02), and increased eschar volume (*P* < 0.001).

**Conclusion:**

LAFA is a useful adjunct for intra-operative stump perfusion assessment and can predict areas of poor stump healing and eschar formation. Diabetic patients seem to be at higher risk of stump eschar formation.

## Introduction

More than 130,000 major lower extremity amputations are performed in the United States each year for critical limb ischemia (CLI).^1^ In up to 40% of patients, poor amputation site healing is associated with surgical site necrosis, dehiscence, and infection, often requiring stump revision and/or re-amputation.^1,2^ The consequences of re-intervention in these highly vulnerable patients is associated with increased disability, morbidity, mortality, and healthcare costs.^3,4^

Conventional modalities for pre-operative assessment of lower extremity arterial inflow and outflow include ankle-brachial index (ABI) with or without lower extremity segmental pressures, transcutaneous oxygen measurement (tcPO_2_), or contrast-based angiography. These diagnostic tools are sometimes used to predict the level at which a below knee amputation (BKA) or above knee amputation (AKA) stump is likely to heal.^5,6^ However, tcPO2 does not provide any specific information about where post-operative wound complications may occur,^7^ and ABIs only have a modest performance in predicting wound healing following surgical intervention.^8^ It is evident that specific patient populations are particularly prone to poor wound healing, and segments of an amputation stump may develop poor healing even in the setting of adequate arterial inflow to the surgical site.^9–11^ These limitations suggest that a ‘real-time,’ intra-operative approach for assessing stump arterial perfusion at the time of amputation can potentially help identify patients who at risk of poor stump healing.

Laser-assisted fluorescence angiography (LAFA) with the SPY Elite^®^ imaging system (Novadaq, Bonita Springs, FL) is an FDA-approved non-invasive imaging modality that utilizes intravenous florescent agents such as indocyanine green dye (ICG) to evaluate tissue perfusion. The clinical utility of LAFA was previously demonstrated in liver, cardiac, and intestinal surgery.^12–14^ LAFA has also been shown to predict viability of mastectomy reconstruction flaps.^15,16^ Despite this, there are only a few reports of LAFA use in patients with lower extremity peripheral arterial disease.^17^ In this study, we retrospectively evaluated whether intra-operative LAFA can identify specific areas of amputation stump mal-perfusion and if these areas are predictive of surgical site healing. We hypothesized that amputation stumps with LAFA-detected mal-perfusion will more likely exhibit signs of prolonged post-operative healing.

## Methods

### Patients

From February to November 2016, all patients who received a major lower extremity amputation (AKA or BKA) and intra-operative LAFA by a single surgeon at our institution were included in the study analysis. Amputation and LAFA were performed as clinically indicated in patients with critical limb ischemia (CLI) who consented for both procedures after a detailed discussion of the risks and benefits. The amputation level (AKA vs BKA) was determined based on the extent of arterial inflow as demonstrated by pre-operative ABIs, cross-sectional angiographic imaging, and the surgeon’s preference. Perioperative patient demographics, comorbidities, and medications were collected and evaluated. Patients were classified as being diabetic based on a review of their past medical history, clinician notes, and usage of anti-glycemic medications (metformin, insulin, thiazolidinediones, etc.).

### Operative technique

AKA and BKA were performed as previously described.^18,19^ Briefly, AKA was performed using a fish-mouth incision in the distal thigh. Following transection of the proximal patellar tendon, the underlying distal femur was circumferentially exposed to the mid-thigh level and transected with a power saw. The femoral vein and superficial femoral artery were each identified at the mid-thigh level within the adductor canal, clamped, transected, and ligated. The sciatic nerve in the posterior compartment was also externalized and transected proximally. The ventral and posterior muscle compartments were then fashioned to facilitate closure, and were tightly approximated over the transected femur with multiple interrupted sutures. Ventral and posterior fascial edges were then approximated also using interrupted sutures, and the transected patellar tendon was used to reinforce the fascial closure. Skin edges were typically closed using simple interrupted sub-dermal sutures, and vertical mattress interrupted dermal sutures or skin staples.

BKA was performed using a long posterior flap technique.^18^ Ventral leg incision was made approximately 10 cm distal to the tibial tubercle and posterior incision long enough to ensure adequate flap coverage. The tibia was transected in a smooth slanted fashion 5 cm proximal to the level of the ventral incision. The fibula was also transected at this proximal level. Each tibial vessel was identified, ligated, and transected. Tibial nerves were externalized and transected proximally. Soleal muscle tissue was flapped over the transected tibia using interrupted sutures, and fascial, sub-dermal, and dermal closures were performed similar to AKA.

### Intra-operative LAFA

Following amputation stump closure and prior to application of sterile dressings, the SPY Elite^®^ imaging system mobile arm was placed 15 cm from the amputation stump to entirely visualize it with the imaging system. The patient’s body temperature was maintained at 36.5–37.5°C. Indocyanine Green (ICG; 7.5 mg in 3 mL solution) was administered through a peripheral intravenous line and flushed with 10 mL of saline. Immediately following ICG administration, the fluorescence intensity of ICG at the amputation stump was recorded with SPY Elite^®^ imaging system according to the manufacturer’s instructions and as previously described,^17^ and continued for a total of 180 s following the initiation of image recording. Stump fluorescence densitometry measurements were analyzed using SPY-Q imaging software and ImageJ. Perfusion intensity was measured for the whole stump and along the suture line. Whole stump perfusion is evaluated as “Integrated Density” of the image, which provides a sum of pixel depth over the selected area. Segmental suture line perfusion measurements were expressed as a ratio relative to an equally sized segment with the highest stump perfusion intensity, thereby normalized to each patient.

### Amputation stump anthropometric analysis

Post-operatively, patients were evaluated periodically for a 4–8-week period. A modified Bates-Jensen Score (mBJS) wound assessment tool (Table 1) was used to assess stump skin color, epithelialization, amount of exudate, and the presence and volume of eschar.^20^ Eschar volume was determined by an observer masked to the intra-operative LAFA using Image J software. Aggregate mBJS were derived for both the whole stump and suture line segments. Wound healing features were evaluated on a 1 to 5 scale system, with lower scores indicating best healing, and higher scores indicating poorest healing. Temporal stump healing was evaluated from stump photographs obtained periodically during the entire post-operative follow-up period. Eschar free period (EFP) and eschar healing period (EFP) were evaluated for each amputation stump.

### Statistical analysis

Patient demographics, stump LAFA perfusion parameters, and anthropometric analysis were performed with GraphPad Prism. Statistics were summarized as mean ± SEM. Non-parametric Spearman correlation analysis was used to determine the relationship mBJS and LAFA perfusion. Mann-Whitney tests were used to determine any differences between diabetic and non-diabetic patients. *P* = 0.05 was considered significant.

### Ethics

Pre-operatively, patients were informed of the risks, benefits, and alternatives of the procedures. The FDA-approved LAFA procedure was described as part of the routine operative and clinical practice of the surgeon performing the amputation to assess intra-operative amputation stump perfusion. This study was approved by the local Institutional Review Board (IRB).

## Results

**Fig. 1:**
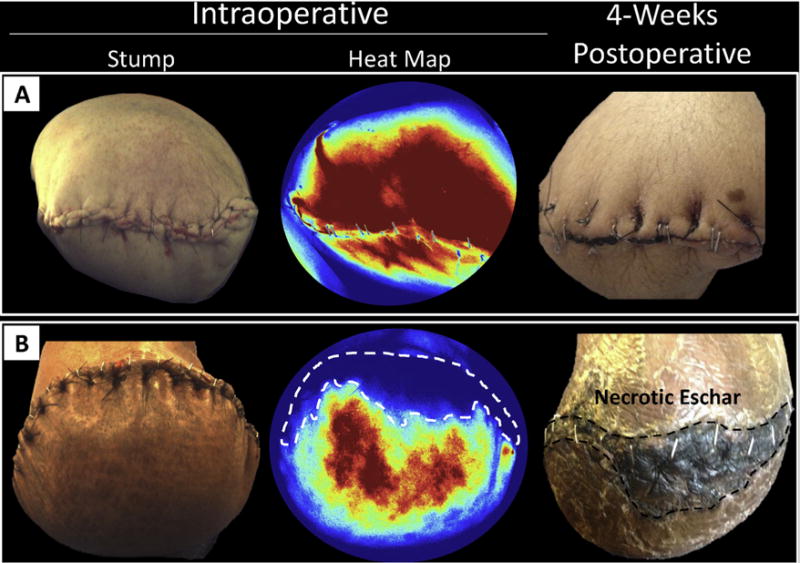
Intra-operative LAFA following amputation stump closure A) A patient demonstrating robust LAFA inflow perfusion as demonstrated on intra-operative perfusion heat map. Post-operatively, this patient demonstrated excellent amputation stump healing. B) Another patient demonstrating relatively less overall stump perfusion, with a large ventral stump perfusion defect (white dashed line). Post-operatively, this perfusion defect correlated with a large eschar formation (black dashed line).

Over the study period, we identified 15 patients undergoing major lower extremity amputation (10 AKAs and 5 BKAs; Table 2). Intra-operative LAFA and post-operative stump surveillance was feasible for all patients (Fig. 1). There were no side effects or complications related to LAFA. Notably, 60% of patients were diabetic, 60% were recent/active cigarette smokers, and 53.3% had significant cardiovascular comorbidities (Table 2). Average ASA classification was 3.3 ± 0.1, consistent with the population’s severe systemic disease.

**Fig. 2:**
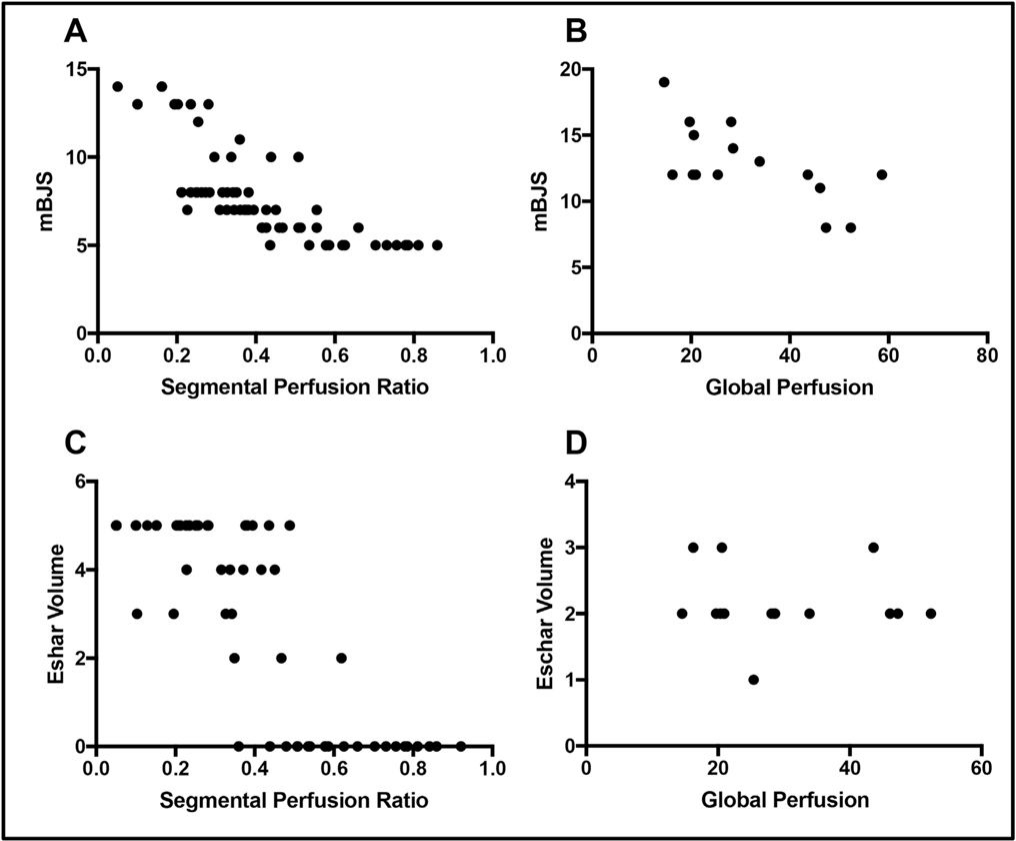
Spearman Correlation Between Amputation Stump Segmental or Global Perfusion and Wound Healing A) Segmental and B) global stump perfusion negatively correlated with mBJS (*P* < 0.001 and *P* = 0.01, respectively). C) Segmental perfusion also correlated negatively with eschar volume (*P* < 0.001); however, global stump perfusion (D) did not demonstrate a significant correlation (*P* = 0.64).

LAFA-derived perfusion demonstrated an inverse correlation to mBJS at the stump suture line (Table 3 and Fig. 2A), and globally on the whole stump surface (Table 4 and Fig. 2B). Perfusion also significantly correlated with the eschar volume at the stump suture line (r = −0.79, CI −0.87 to 0.67, *P* < 0.001; Fig. 2C). Global stump perfusion did not correlate with any wound parameters on the stump surface (Table 4; Fig. 2D), indicating that stump perfusion mostly affected healing at the suture line.

**Fig. 3:**
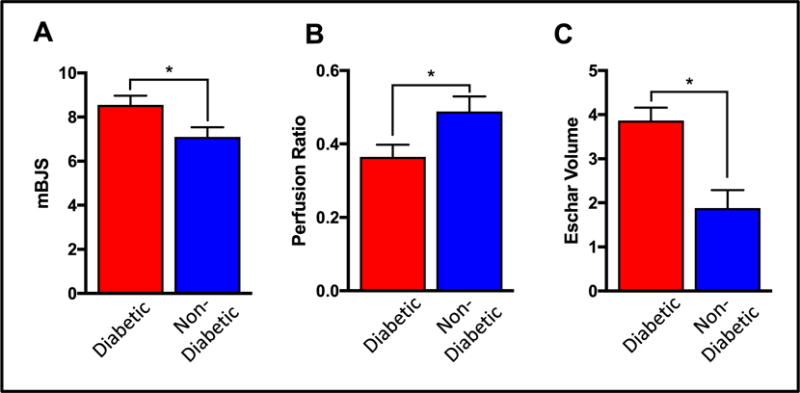
Assessment of segmental amputation stump perfusion and healing in diabetic and non-diabetic patients A) Diabetic patients demonstrated significantly higher segmental mBJS and (B) lower segmental perfusion than non-diabetic patients. C) Diabetic patients also had significantly larger eschars than non-diabetic patients. (*P < 0.05).

**Fig. 4:**
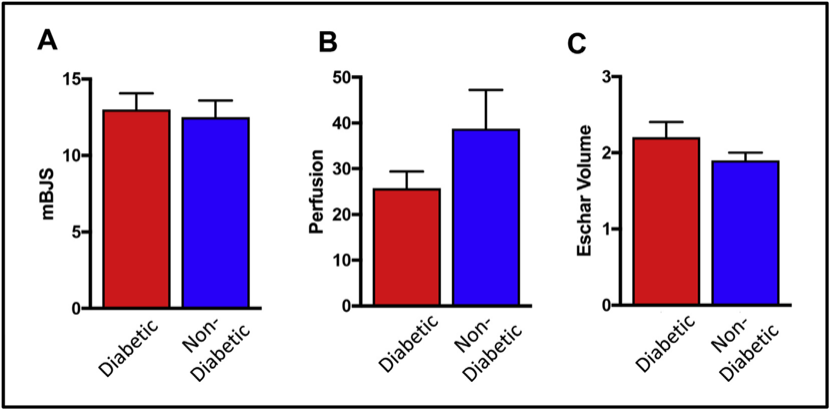
Assessment of global amputation stump perfusion and healing in diabetic and non-diabetic patients We found no significant differences between diabetic and non-diabetic patients in (A) mBJS, (B) perfusion, and (C) eschar volume.

**Fig. 5:**
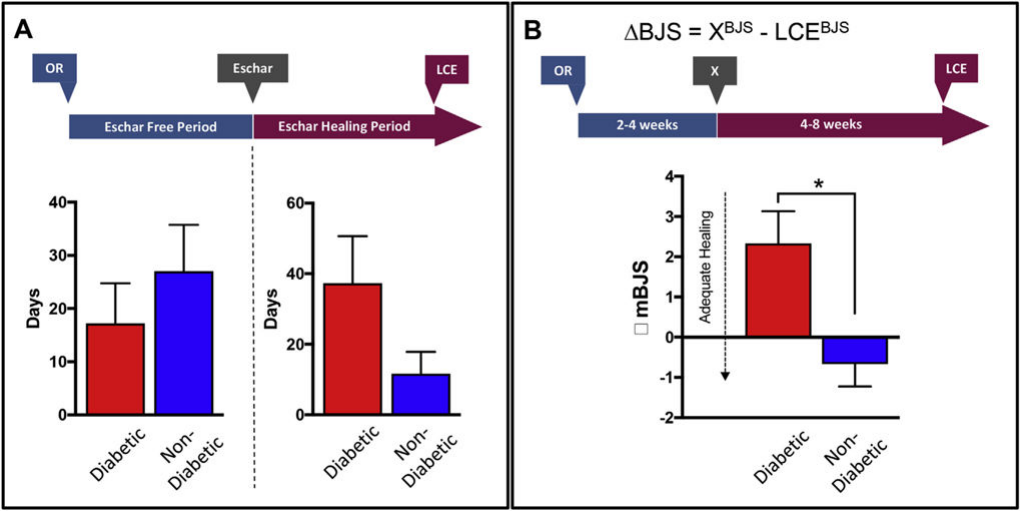
Temporal amputation stump healing in diabetic VS non-diabetic patients A) We observed no significant differences in eschar free and escharing healing periods between diabetic and non-diabetic patients, though diabetics tended to have shorter eschar free periods and longer eschar healing periods. B) Change in mBJS scores between 4 weeks of healing (time point X) and time of last clinical evaluation (LCE, 4–8 weeks after operation), demonstrated less adequate healing in diabetic patients compared to non-diabetics (*denotes significant value).

Since 60% of the study cohort was diabetic, we evaluated whether diabetics exhibited differences in global and segmental stump perfusion, and suture line healing. Average hemoglobin A1C for the diabetic patients was 6.8 ± 0.5%. Diabetic patients had higher mBJS (*P* = 0.009; Fig. 3A), lower segmental suture line perfusion (*P* = 0.02; Fig. 3B), and larger eschar volume at the suture line (*P* < 0.001, Fig. 3C). Diabetic patients also demonstrated non-significant trends in lower global stump perfusion (Fig. 4B), and larger eschar volume (Fig. 4C). Diabetic patients also demonstrated non-significant trends in shorter EFPs and longer EHPs (Fig. 5A). No differences were observed in stump wound free or wound healing periods between diabetic and non-diabetic patients (Supplemental Fig. 1). However, over the post-operative recovery period, diabetic patients demonstrated a significant relative increase in mBJS (ΔBJS; Fig. 5B), consistent with poorer wound healing over time in this patient group.

## Discussion

In this study, we demonstrate that LAFA is a useful intra-operative adjunct test that can predict areas of amputation stump mal-perfusion and subsequent poor healing. We also demonstrate that adequate perfusion at the suture line is important for avoiding post-operative eschar formation. Furthermore, diabetic patients appear to be more at risk of amputation stump suture line eschar formation and delayed healing.

Unlike the majority of patients requiring limb amputation due to trauma or malignancy, patients who receive an amputation as a result of end-stage CLI are much more prone to developing poor healing, infection, and need for re-intervention at the amputation site.^21^ In addition, it is not uncommon that patients undergoing major lower extremity amputation for CLI have already endured prior periods of pain, infection, and attempted revascularization.^22^ Some studies have cited stump breakdown and dehiscence rates of 16%, with infection rates of 22%,^23^ and ischemia being a significant contributor to both complications.^24^

A number of previous studies have evaluated the morbidity and mortality associated with lower extremity amputation. A study of 716 patients who received both AKAs or BKAs were found to have a wound infection rate of 5.5%, re-intervention rate of 3.2%, stump revision rate of 2.3%, and BKA to AKA conversion rate of 9.4%.^25^ Other studies have also demonstrated a BKA to AKA conversion rate of 9–20%.^25–28^ This suggests that not infrequently, amputation stumps can develop areas of significant poor healing leading to reoperation, amputation stump revision, or re-amputation at a higher level. Patients with AKAs are thought to have better arterial inflow; however still may require surgical revision due to poor healing.^25^ These studies also suggest that current pre-operative diagnostic modalities with ABIs, contrast based angiography, and tcPO2 may provide some insight into assessing the appropriate level of amputation, though the number of revisions and re-interventions suggests that a more predictive, dynamic, and intra-operative imaging modality may be required for optimizing predictive wound healing in these patients.

Our study suggests that intra-operative LAFA stump assessments may be a useful tool in addition to conventional methods. LAFA with the SPY Elite^®^ imaging system can be performed within the sterile operative field, and provide both qualitative as well as quantitative perfusion assessments. The quantitative variables can provide relative comparisons between different studies at different time points, and help derive perfusion thresholds for adequate tissue healing. Since we observed that suture line perfusion is an important indicator of likelihood of eschar formation and stump healing, future use of intra-operative LAFA can provide a surgeon the opportunity to revise the amputation stump closure, and/or post-operatively closely survey the area of mal-perfusion. To this effect, surgeons could also consider performing LAFA on the amputation skeletal muscle tissue flaps prior to closure, or perform a temporary stump closure to assess the perfusion of the skin closure. This may allow for an opportunity to revise the amputation stump if necessary before the final closure. Additionally, we observed that LAFA may provide the surgeon and patient the opportunity to start with a BKA in a scenario where healing may be questionable based on pre-operative testing. Depending on the LAFA study findings, the surgical plan may be revised in real-time. Similar to others, we have found that the use of intra-operative LAFA is rapid and non-disruptive to the flow of the operation.^29^

Previous studies have demonstrated decreased wound healing in diabetic patients.^30,31^ Similarly, our study demonstrates delayed suture line healing in diabetic patients undergoing lower extremity amputation. This is despite adequate glycemic control in the diabetic patient cohort included in our study, and diabetic patients having an average hemoglobin A1c of 6.8 ± 0.5%. A possible explanation for this observation is the relatively decreased global and segmental suture line perfusion of amputation stumps in diabetic patients (Figs. 4 and 5). Less arterial inflow and oxygenation of the healing stump can potentiate hypoxia at the surgical site, increase free-radical formation, and lead to tissue damage and eschar formation.^32,33^ Early detection of relative stump mal-perfusion in at risk diabetic patients can help inform the post-operative surveillance time-line and anticipated wound care needs.

Unlike studies that report readmission rates of 26.3% for patients who have undergone major lower extremity amputations,^34,35^ the 30-day re-admission rate for our cohort was only 13.3% (Supplemental Fig. 1). Patients with reduced perfusion as evidenced by their intra-operative assessment were discharged from the hospital with arrangements for wound care assessments and nursing, as well as regular bi-weekly outpatient clinical evaluations by the surgical team. A future topic of investigation will focus on the use of intra-operative LAFA to determine specific post-operative care needs to reduce hospital readmissions and re-operations.

We acknowledge that there are limitations to our single-center cohort study. One limitation is the small sample size, which can introduce bias into the statistical evaluation of the cohort; however, we determined that our study findings are consistent with other recent reports that have utilized LAFA in other contexts.^12–14,17^ Despite technical limitations of the SPY Elite apparatus, which restricts the imaging of only one anatomical region at a time, and tissue penetration of 3 mm, LAFA imaging was still informative and provided adjunct information that correlated with post-operative healing parameters. We believe this pilot study demonstrates the technical feasibility of performing LAFA in lower extremity amputation patients and demonstrates the ability of LAFA to predict intra-operatively where wound breakdown and inadequate healing is likely to occur. To this degree, this may offer surgeons an intra-operative opportunity to revise an amputation to a higher level, or if necessary inform the care team to maintain increased post-operative stump survillance.

## Conclusions

Our study demonstrates that intra-operative LAFA can provide perioperative adjunct parameters that can help predict amputation stump healing. Patients with decreased LAFA perfusion along the suture line are more likely to develop post-operative necrotic eschars. Even ‘well-controlled’ diabetic patients are more prone to delayed stump healing and eschar formation. Future studies may focus on adjunct therapies that can augment amputation stump perfusion in at risk patients to enhance surgical site healing.

## Supplementary Material

1

2

3

## Figures and Tables

**Table 1:** mBJS for amputation stump healing.

Necrotic Tissue Type	1 = None
	2 = Non-adherent slough
	3 = Adherent slough
	4 = Soft eschar
	5 = Hard eschar
Necrotic Tissue Volume	1 = None
	2 = < 25% of wound bed
	3 = 25-50%
	4 = 50-75%
	5 = 75-100%
Exudate Type	1 = None
	2 = Bloody
	3 = Serosanguinous
	4 = Serous
	5 = Purulent
Skin Color Surrounding Wound	1 = Pink
	2 = Bright red
	3 = Hypopigmented
	4 = Dark red
	5 = Hyperpigmented
Epithelialization	1 = 100% of wound
	2 = 75-100%
	3 = 50-75%
	4 = 25-50%
	5 = < 25%

**Table 2:** Study cohort demographics and comorbidities.

Demographics	Mean ± SEM
Age (years)	63.9 ± 2.2
Gender	Males (n = 9)
	Females (n = 6)
ASA	3.3 ± 0.1
BMI	24.6 ± 1.4
**Amputation**	***N* (%)**
Below Knee	5 (33.3%)
Above Knee	10 (66.7%)
Previous Contralateral Limb	1 (6.7%)
**Comorbidities**	***N* (%)**
Smoking	9 (60%)
Hypertension	11 (73.3%)
Hyperlipidemia	7 (46.7%)
Diabetes	9 (60%)^a^
Coronary Artery Disease	8 (53.3%)

a Average hemoglobin A1c - 6.8 ± 0.5%; Range 4.8–10.3%.

**Table 3:** Spearman correlation analysis of segmental perfusion and wound healing.

Segmental Perfusion	R	CI	P
mBJS	−0.87	−0.91 to −0.79	<0.001
Eschar Score	−0.81	−0.89 to −0.72	<0.001
Eschar Volume Score	−0.79	−0.87 to −0.67	<0.001
Exudate Score	−0.32	−0.67 to 0.51	0.13
Skin Color Score	−0.11	−0.48 to 0.63	0.60
Epithelialization Score	−0.46	−0.76 to 0.10	0.12

**Table 4:** Spearman correlation analysis of global perfusion and wound healing.

Global Perfusion	R	CI	P
BJS	−0.62	−0.87 to −0.15	0.01
Eschar Score	−0.32	−0.72 to 0.25	0.25
Eschar Volume Score	−0.13	−0.62 to 0.42	0.64
Exudate Score	−0.23	−0.67 to 0.34	0.48
Skin Color Score	−0.46	−0.79 to 0.081	0.10
Epithelialization Score	−0.43	−0.78 to 0.12	0.13

## Appendix A. Supplementary data

Supplementary data related to this article can be found at https://doi.org/10.1016/j.amjsurg.2018.05.007.
