# Supplementary figures and images for: Amputation stump perfusion is predictive of post-operative necrotic eschar formation
Source: Am J Surg. Author manuscript; Available in PMC 2019 Sep 1. (PMC6129216; doi:10.1016/j.amjsurg.2018.05.007)

## Slide 1
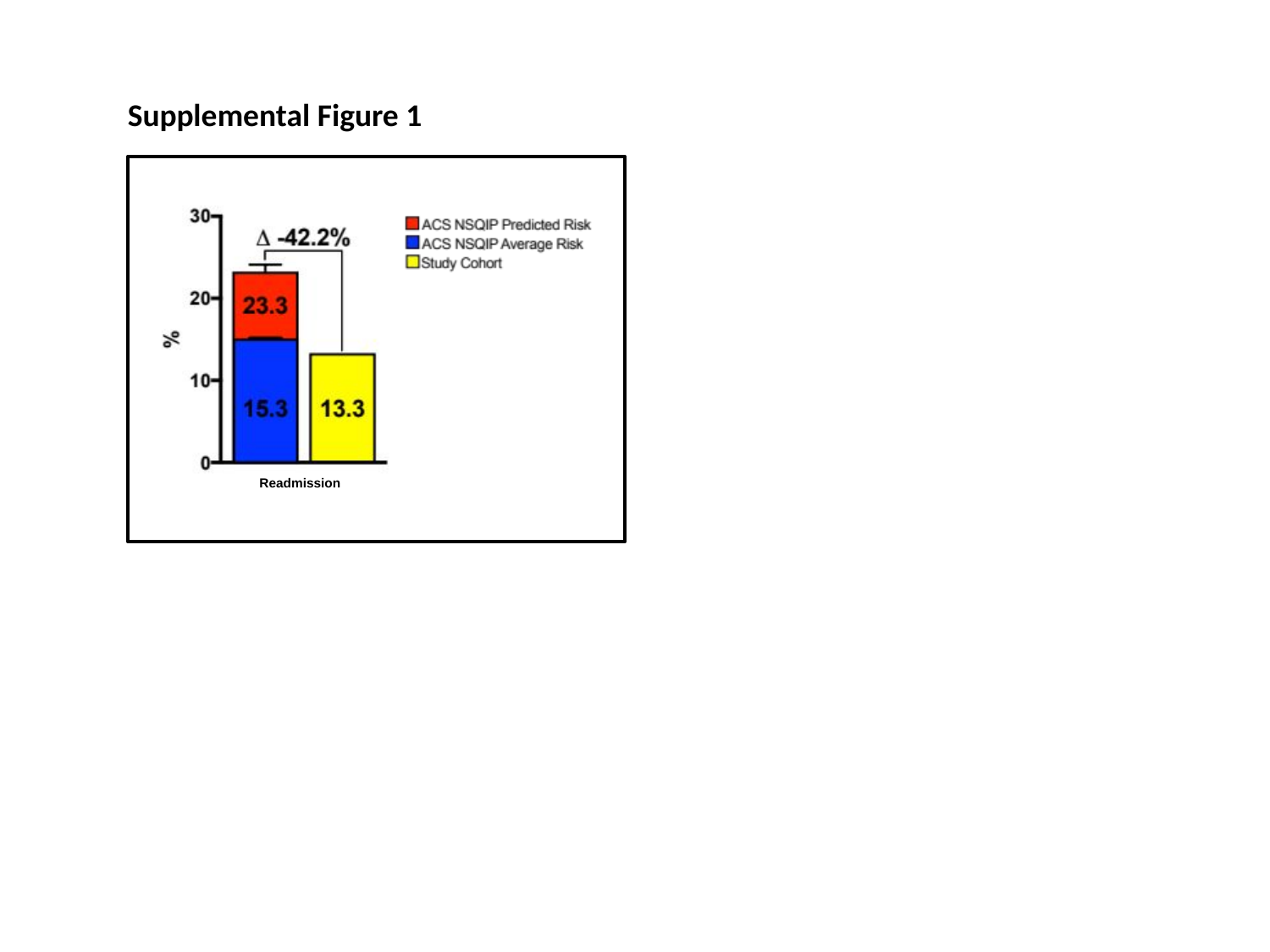

Supplemental Figure 1
Readmission

Supplement: 2 [file NIHMS967208-supplement-2.pptx]
