# Supplementary material for: Amputation stump perfusion is predictive of post-operative necrotic eschar formation
Source: Am J Surg. Author manuscript; Available in PMC 2019 Sep 1. (PMC6129216; doi:10.1016/j.amjsurg.2018.05.007)
Supplement: 3 [file NIHMS967208-supplement-3.pptx]

## Slide 1
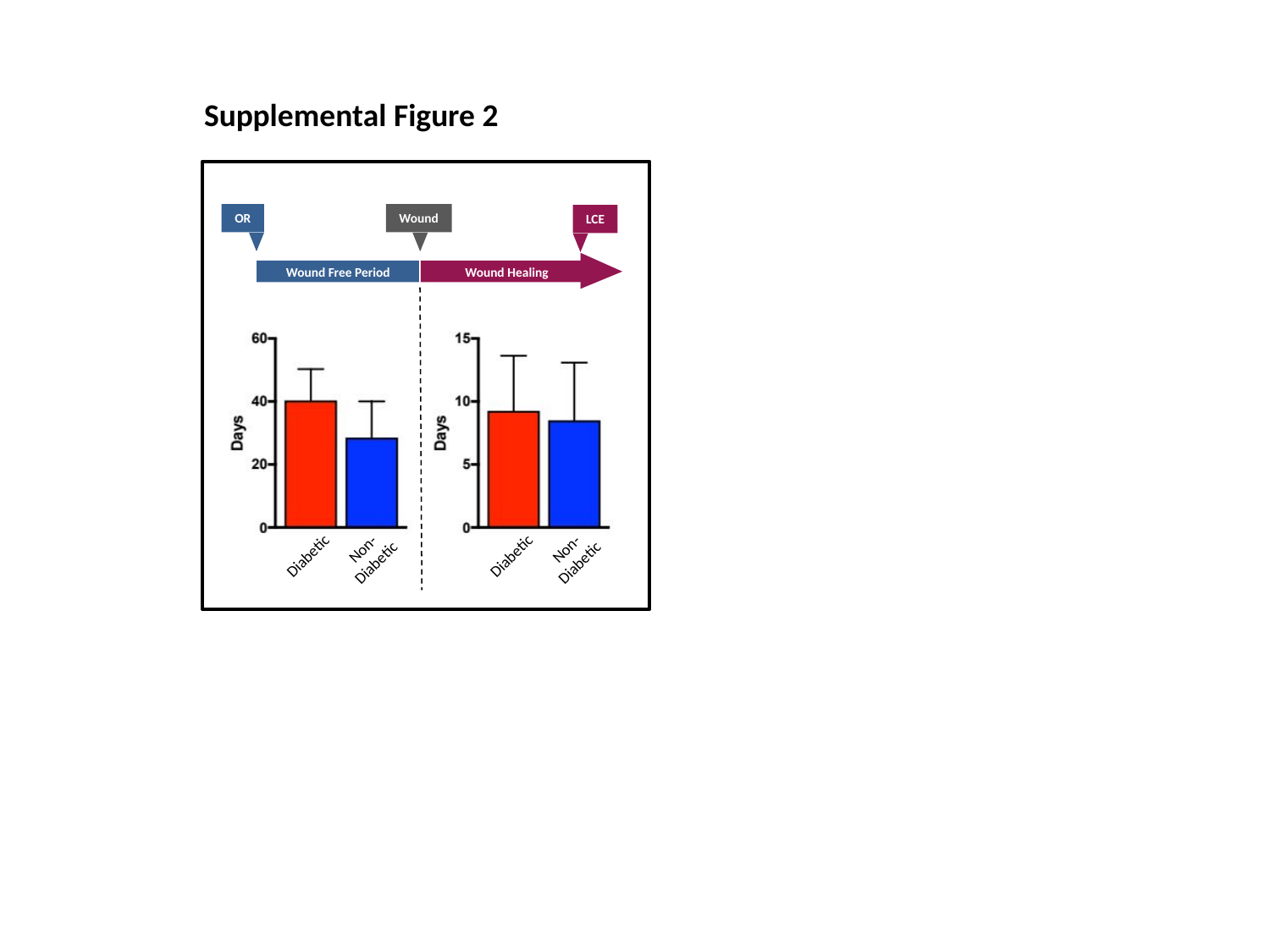

Supplemental Figure 2
Wound
OR
LCE
Wound Free Period
Wound Healing Period
Non-Diabetic
Non-Diabetic
Diabetic
Diabetic
